# The cell cycle regulator p16 promotes tumor infiltrated CD8^+^ T cell exhaustion and apoptosis

**DOI:** 10.1038/s41419-024-06721-7

**Published:** 2024-05-15

**Authors:** Xin Zhang, Jiajia Wang, Kun Tang, Yu Yang, Xiaowei Liu, Shengtao Yuan, Feng Guo, Lianjun Zhang, Kaili Ma

**Affiliations:** 1 National Key Laboratory of Immunity and Inflammation, Suzhou Institute of Systems Medicine, Chinese Academy of Medical Sciences & Peking Union Medical College, Suzhou, Jiangsu China; 2 Key Laboratory of Synthetic Biology Regulatory Element, Suzhou Institute of Systems Medicine, Chinese Academy of Medical Sciences and Peking Union Medical College, Suzhou, China; 3 Institute of Pharmaceutical Sciences, China Pharmaceutical University, Nanjing, Jiangsu China; 4 Institutes of Biology and Medical Sciences (IBMS), Soochow University, Suzhou, Jiangsu China; 5 Department of Oncology, The Affiliated Suzhou Hospital of Nanjing Medical University, Suzhou, Jiangsu China

**Keywords:** Immune evasion, Immune cell death

## Abstract

The therapeutic efficacy of adoptive T cell therapy is largely restricted by reduced viability and dysfunction of CD8^+^ T cells. Continuous antigen stimulation disrupts the expansion, effector function, and metabolic fitness of CD8^+^ T cells, leading to their differentiation into an exhausted state within the tumor microenvironment (TME). While the function of the cell cycle negative regulator p16 in senescent cells is well understood, its role in T cell exhaustion remains unclear. In this study, we demonstrated that TCR stimulation of CD8^+^ T cells rapidly upregulates p16 expression, with its levels positively correlating with TCR affinity. Chronic TCR stimulation further increased p16 expression, leading to CD8^+^ T cell apoptosis and exhaustion differentiation, without inducing DNA damage or cell senescence. Mechanistic investigations revealed that p16 downregulates mTOR, glycolysis, and oxidative phosphorylation (OXPHOS) associated gene expression, resulting in impaired mitochondrial fitness, reduced T cell viability, and diminished effector function. Furthermore, the deletion of p16 significantly enhances the persistence of CD8^+^ T cells within tumors and suppresses the terminal exhaustion of tumor-infiltrating T cells. Overall, our findings elucidate how increased p16 expression reshapes T cell intracellular metabolism, drives T cell apoptosis and exhaustion differentiation, and ultimately impairs T cell anti-tumor function.

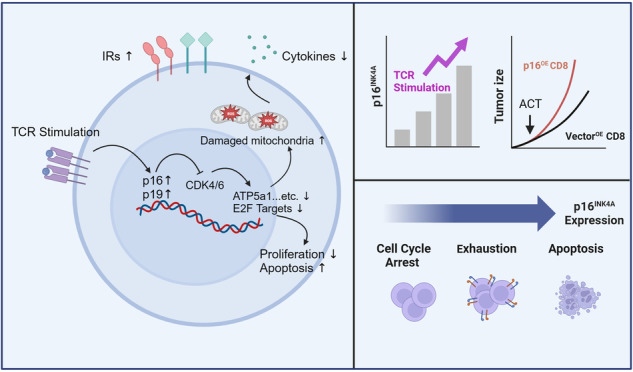

## Introduction

Cytotoxic CD8^+^ T cells play a critical role in eliminating tumor cells by producing Granzyme B (GzmB) and effector cytokines such as IL-2, TNF-α, and IFN-γ [1]. The persistence and differentiation status of the tumor antigen­specific CD8^+^ T cells determine the efficacy of anti-tumor immunotherapy [2]. However, prolonged antigen exposure leads to the gradual differentiation of tumor infiltrating CD8^+^ T cells into an exhausted state, which is characterized by impaired proliferation and effector function, along with increased expression of various inhibitory receptors (e.g., PD-1 and TIM-3, etc.) [3]. Emerging evidence indicates that exhausted CD8^+^ T cells within the tumor microenvironment (TME) harbor significant metabolic defects, including mTOR signaling inhibition, impaired mitochondrial activity, reduced glycolysis, and decreased oxidative phosphorylation levels [4–7].

Beyond exhaustion, metabolic competition with regulatory T (Treg) cells triggers evident DNA damage and senescence in CD8^+^ T cells, ultimately leading to limited immunotherapeutic efficacy [8]. Senescence and exhaustion states of T cells have overlapping characteristics and are associated with defects in effector functions [9, 10]. Upregulation of cell cycle regulatory genes such as *p16*, *p21*, and *p53* results in cell cycle arrest and has been identified as a cardinal feature of senescent cells [11]. Specifically, p16, a protein encoded by the *Cdkn2a* gene, binds to the cell cycle-dependent kinase 4/6 (CDK4/6) and inhibits its catalytic activity [12]. CDK4/6, in association with cyclin D1 proteins, phosphorylates retinoblastoma proteins (RB) and releases RB from E2F. The transcription factor E2F is responsible for the expression of genes required for the cell cycle transition from the G1 to S phase [13].

The expression and role of p16 in T cell development and differentiation have attracted considerable attention. Previous studies have shown that overexpression of p16 in thymocytes inhibits T cell development at the double-negative (DN) stage [14]. Recently, Janelle et al. found that DNA damage could induce p16 expression in tumor-infiltrating exhausted CD8^+^ T cells, leading to impaired cell cycle progression. Furthermore, they found that knockdown of p16 could enhance the functionality of exhausted CAR-T cells in vitro [15]. However, there is currently a lack of in vivo evidence to fully understand the regulatory role of p16 expression in the exhaustion of tumor-infiltrating CD8^+^ T cells. Additionally, the administration of CDK4/6 inhibitor Palbociclib has been shown to promote CD8^+^ T cell differentiation into memory-like phenotypes, thereby bolstering the anti-tumor effects of adoptive T cell therapy [16]. Therefore, the significance of cell cycle arrest in determining the differentiation state of CD8^+^ T cells within the TME remains largely undefined, and further investigation is needed to clarify the precise role of the cell cycle regulator p16 in CD8^+^ T cell exhaustion.

Here, we confirmed the dynamic expression of p16 in CD8^+^ T cells following TCR stimulation and observed a rapid increase in p16 levels upon TCR stimulation in vitro. Overexpression of p16 and another isoform of the *Cdkn2a* gene, p19, in T cells inhibited CD8^+^ T cell proliferation and promoted apoptosis rather than inducing senescence or causing cellular DNA damage. Specifically, the upregulation of p16 impaired the mitochondrial metabolism and fitness, resulting in T cell exhaustion and the suppression of effector function, attributable to the inhibition of CDK4/6 kinase. Elevated expression of p16 resulted in the differentiation of adoptively transferred CD8^+^ T cells into terminal exhaustion and reduced T cell persistence in vivo. Conversely, the deletion of p16 partially reduced mito-ROS production and attenuated the terminal exhaustion of CD8^+^ T cells both in vitro and in vivo. Therefore, our study revealed that the upregulation of p16 is an early event of T-cell dysfunction in the TME. Elevated p16 levels further impedes the survival capacity of CD8^+^ T cells and the promoted terminal exhaustion by remodeling cellular metabolism, suggesting that cell cycle arrest might play a critical role in reinforcing the exhaustion state of tumor-initiated T cells.

## Results

### Rapid upregulation of p16 in CD8^+^ T cells following TCR stimulation

**Fig. 1:**
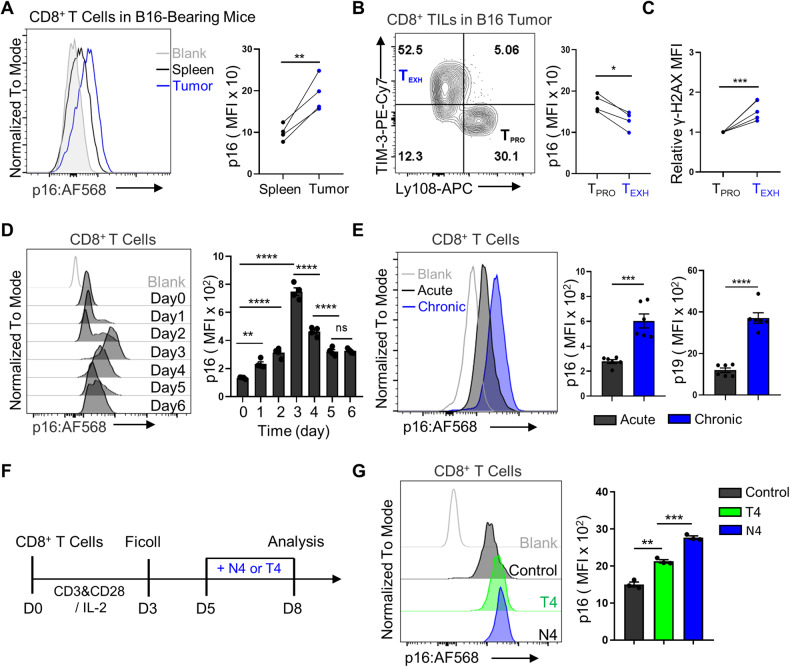
The dynamic expression of p16 in CD8^+^ T cells. **A** Left: A representative histogram displays p16 expression in spleen or B16-F10 tumor-infiltrated CD8^+^ T cells. Right: Statistical analysis of p16 mean fluorescence intensity (MFI) in the indicated cells. Data are presented as mean ± SEM (*n* = 4), Student’s *t* test, **P *<* 0.01. **B**, **C** Left: CD8^+^ TILs were segregated into Ly108^+^TIM-3^-^ T_PRO_ and Ly108^-^TIM-3^+^ T_EXH_ in contour plots. Right: Comparison of p16 and γ-H2AX levels in these two subsets. Data are shown as mean (*n* = 4), Student’s *t* test, *P < 0.05; ***P *<* 0.001. **D** Left: Representative histogram depicting p16 expression in CD8^+^ T cells. Right: Quantification of the dynamic changes in p16 expression in OT-1 cells during OVA peptide activation. Data are shown as mean ± SEM (*n* = 3), one-way ANOVA, **P *<* 0.01; ****P < 0.0001; ns not significant. **E** Left: Activated OT-1 cells were re-stimulated with or without OVA peptide continuously for 4 days, designated as chronic or acute treatment. Representative FACS plots illustrating p16 expressions are displayed. Right: Flow cytometry analysis quantified the MFI of p16 and p19. Data are shown as mean ± SEM (*n* = 6), Student’s *t* test, ***P *<* 0.001; ****P < 0.0001. **F** Experimental design for investigating p16 expression in response to various TCR stimulations. **G** Activated OT-1 cells were exposed to either high-affinity (N4) or low-affinity (T4) OVA peptides for 3 days. Representative FACS plots illustrating p16 expression are provided (left). Flow cytometry analysis was used to determine the MFI of p16 and summarize the data (right). Data are shown as mean ± SEM (*n* = 3), Student’s *t* test, **P *<* 0.01; ***P < 0.001.

The exhaustion of tumor-infiltrating CD8^+^ T cells with poor proliferation capacity greatly restricts the efficacy of immunotherapy. To investigate the specific function of the cell cycle regulator p16 during CD8^+^ T cell exhaustion, we first compared the expression pattern of p16 in tumor-infiltrating T cells (TILs) and T cells resident in the spleen. We found that p16 is upregulated in tumor-infiltrating T cells, including those in mouse melanoma cancer (B16) and colorectal cancer (MC38) models (Fig. 1A and Supplementary Fig. S1A). Further real-time PCR analysis of spleen and tumor infiltrating T cells confirmed that the transcription levels of p16 or p19 were indeed significantly elevated in CD8^+^ TILs (Supplementary Fig. S1B). These results indicated that p16 and p19 may be involved in the exhaustion differentiation of CD8^+^ T cells. Interestingly, we observed that the levels of p16 in Ly108^+^TIM-3^−^ progenitor exhausted T cells were higher than in Ly108^-^TIM-3^+^ terminally exhausted T cells (Fig. 1B). Given the persistent TCR stimulation-induced DNA damage of T cells [17], which could activate the signal transduction pathway of p16-RB and upregulate the expression of p16 [18, 19], we thus investigated DNA damage in both progenitor and terminally exhausted populations using γ-H2AX. Surprisingly, the levels of DNA damage were higher in terminally exhausted T cells compared to progenitor T cells, which differed from the expression pattern of p16 (Fig. 1C), suggesting that the expression of p16 is not solely induced by DNA damage.

In addition, we investigated the dynamic expression of p16 in ovalbumin (OVA) peptide-stimulated TCR-transgenic OT-1 cells in vitro and observed the upregulation of p16 levels following antigen stimulation (Fig. 1D). Furthermore, we analyzed the expression of p16 during the differentiation of CD8^+^ T cell exhaustion in vitro (Supplementary Fig. S1C). Flow cytometry and real-time PCR assays revealed a significant increase in p16 and p19 expression in CD8^+^ T cells under continuous TCR stimulation (Fig. 1E and Supplementary Fig. S1D). To determine whether the upregulation of p16 is associated with the intensity of TCR signals, we activated OT-1 cells with two sets of antigen stimulation with distinct affinities, namely OVA peptide N4 with high TCR affinity and T4 with low TCR affinity (Fig. 1F), and observed that p16 expression was further increased along with high-affinity TCR stimulation (Fig. 1G). These findings indicate that p16 can be rapidly upregulated when CD8^+^ T cells respond to TCR stimulation.

### The ectopic expression of p16 or p19 inhibited the proliferation and survival of CD8^+^ T cells

**Fig. 2:**
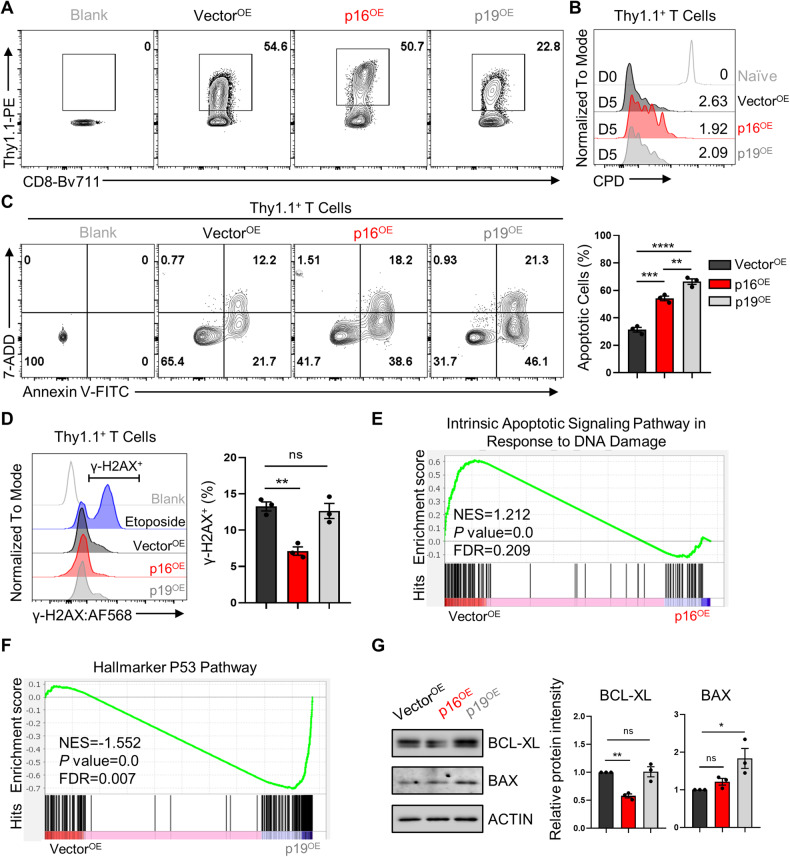
Effects of p16^OE^ or p19^OE^ on CD8^+^ T cell proliferation and survival independent of DNA damage signaling pathway. **A** Representative FACS plots of Thy1.1 showing the infection efficiency of Vector, p16 and p19. **B** Naive OT-1 cells were stained with cell proliferation dye (CPD 450), and their proliferation index was assessed by flow cytometry. **C** Left: Contour plots displaying 7-AAD/Annexin V staining for cell apoptosis. Right: Summary of the relative apoptosis levels of OT-1 cells overexpressing p16 or p19. **D** Left: Representative FACS plots showing γ-H2AX expression. Right: Statistical analysis of γ-H2AX MFI in the indicated cells. Data are shown as mean ± SEM (*n* = 3), one-way ANOVA, *P *<* 0.05; **P *<* 0.01; ***P < 0.001; ns not significant. **E**, **F** Gene set enrichment analyses (GSEA) were conducted to assess the enrichment of gene sets associated with DNA damage and the p53 signature in CD8^+^ T cells overexpressing Vector compared to those overexpressing p16 or p19. Nominal *P* values and false discovery rates (FDRs) were calculated using the default method in the GSEA software. **G** Western blot analyses were performed to assess the protein levels of BCL-XL and BAX in Vector^OE^, p16^OE^, or p19^OE^ OT-1 cells. Right: A comparison of the protein levels of these indicated proteins among the three groups. Data are shown as mean ± SEM (*n* = 3), one-way ANOVA, *P *<* 0.05; **P *<* 0.01; ns not significant.

Given the specific roles of p16 and p19 in cell cycle arrest, we proceeded to assess the proliferation ability and cell survival of T cells upon ectopic expression of p16 and p19. To this end, we overexpressed p16 or p19 in OT-1 cells using a retroviral transduction system containing Thy1.1 as the positive marker. The efficiency of retroviral infection was confirmed by measuring Thy1.1 expression after 24 h of culture (Fig. 2A). mRNA analysis further confirmed the overexpression of p16 and p19 (Supplementary Fig. S2A). Additionally, flow cytometry results using anti-p16 and anti-Thy1.1 antibodies verified that Thy1.1 signals could indicate the overexpression efficiency of p16 (Supplementary Fig. S2B). Indeed, both p16^OE^ and p19^OE^ significantly inhibited CD8^+^ T cell proliferation (Fig. 2B). Consistently, RNA sequencing analysis revealed a significant reduction in the expression of MYC downstream target genes in T cells overexpressing p16 and p19, indicating that the decreased proliferation was likely dependent on MYC signaling (Supplementary Fig. S2C, D).

Additionally, the percentage of apoptotic cells was significantly higher in p16^OE^ and p19^OE^ cells than in the Vector^OE^ group (Fig. 2C), suggesting that both upregulation of p16 and p19 proteins which are encoded by *Cdkn2a* can induce CD8^+^ T cell apoptosis. Moreover, we sorted T cells based on low and high exogenous expression of p16 and found that cells with high p16 expression were more susceptible to death (Supplementary Fig. S1E, F). Further flow cytometric analysis showed that the γ-H2AX^+^ population in p16^OE^ T cells was lower than the control group, whereas p19^OE^ had no effect on the γ-H2AX^+^ population (Fig. 2D). RNA sequencing analysis also revealed that genes associated with DNA damage-induced intrinsic apoptotic signaling were not enriched in CD8^+^ T cells overexpressing p16 or p19 (Fig. 2E and Supplementary Fig. S2E–G). However, T cellsoverexpressing p19 showed enrichment of genes related to the p53 pathway (Fig. 2F and Supplementary Fig. S2H), consistent with previous reports suggesting that p19 relies on p53 to inhibit cell cycle progression [20, 21]. Subsequently, Western blot analysis was conducted to assess the levels of anti-apoptotic protein BCL-XL [22], and pro-apoptotic BAX [23]. The results demonstrated that p16^OE^ significantly reduced the content of anti-apoptotic protein BCL-XL but had no effect on the pro-apoptotic protein BAX, whereas p19 significantly upregulated the expression of BAX (Fig. 2G). Consistent with our results, previous studies have indicated that the ectopic expression of p19 can trigger apoptosis through either p53-dependent or -independent pathways [24, 25]. Additionally, it has been also suggested that there is a suggestion that ectopic expression of p16 into viable K562 cells promoted erythroid differentiation, while in K562 cells with incomplete differentiation, it induced apoptosis. This process is associated with the reduction of BCL-XL and nuclear NF-Κb levels [26]. Consequently, our results imply that the induced apoptosis by either p16 or p19 was not via the DNA damage signaling pathway, but rather by reducing the levels of the anti-apoptotic protein BCL-XL or increasing those of the pro-apoptotic protein BAX.

### The ectopic expression of p16 or p19 was insufficient to induce classical senescence in T cells

**Fig. 3:**
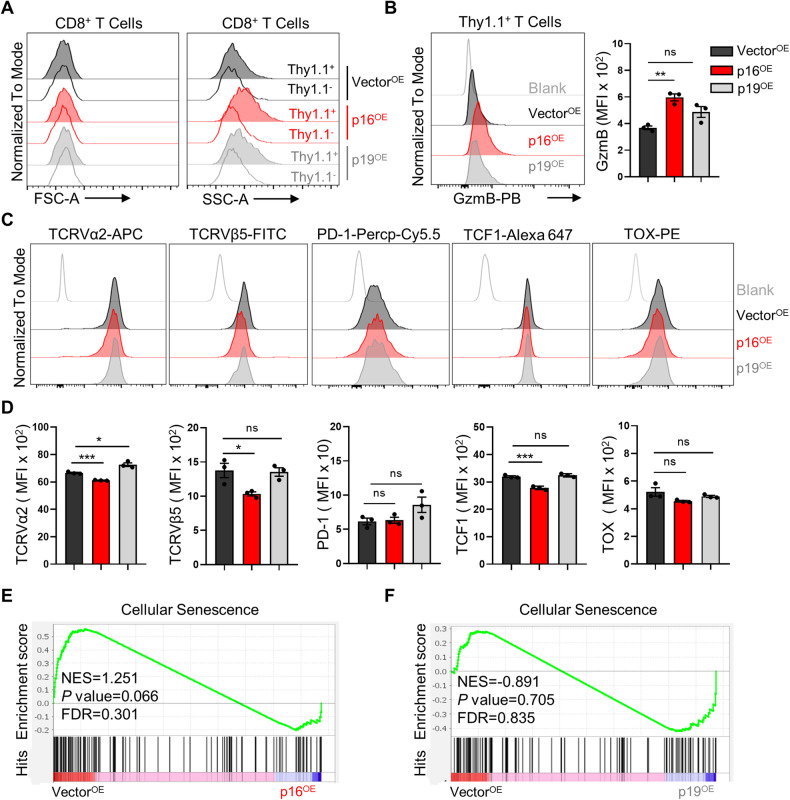
Overexpression of p16 or p19 does not trigger evident CD8^+^ T cell senescence. **A** Naive OT-1 cells were activated by αCD3/CD28 for 2 days, followed by infection with p16 or p19 retrovirus. Three days post-infection, the forward scatter (FSC) and side scatter (SSC) of the specified cells were assessed using flow cytometry. **B** Left: A representative histogram of Granzyme B in Vector^OE^, p16^OE^, p19^OE^ CD8^+^ T cells. Right: Statistical analysis of Granzyme B MFI in indicated cells. **C**, **D** Flow cytometry was utilized to analyze the phenotype of p16^OE^ or p19^OE^ T cells and representative histograms were presented (upper panel). MFI of indicated proteins (TCRVα2, TCRVβ5, PD-1, TCF-1, TOX) in indicated OT-1 cells were summarized (lower panel). Data are shown as mean ± SEM (*n* = 3), one-way ANOVA, *P *<* 0.05; **P *<* 0.01; ***P < 0.001; ns not significant. **E**, **F** Gene set enrichment analysis for cellular senescence was performed using RNA-seq data from Vector^OE^, p16^OE^ p19^OE^ enriched CD8^+^ T cells. Nominal P values and FDRs were calculated with the default method in the GSEA software.

Erickson et al. observed an upregulation of p16 in human T lymphocytes cultured to senescence in vitro [27]. Additionally, Migliaccio et al. found that while silencing p16 expression rescued T cell proliferation, replicative senescence of CD8^+^ T lymphocytes after extended in vitro culture was unrelated to p16 [28]. We thus investigated whether the overexpression of p16 or p19 could induce CD8^+^ T cell senescence. Previous studies have indicated that senescent T cells typically exhibit increased cell size and granularity [29], along with reduced expression of effector molecules such as GzmB and perforin [29]. Additionally, several studies have suggested that aged human T cells may consist of various distinct populations [30–33]. For instance, the CD8^+^CD28^−^ T cells and effector memory T cells expressing CD45RA (Temra) cells have been shown to exhibit activation of p38 mitogen-activated protein kinase (MAPK), secretion of senescence-associated secretory phenotype (SASP), phosphorylation of γ-H2AX, and expression of surface markers associated with senescence such as KLRG1 [30, 31, 34]. The virtual memory T (Tvm) population has been shown to exhibit impaired TCR stimulation [32, 35]. Age-associated CD8^+^ T cells (Taa) are characterized by increased expression of exhaustion markers such as PD-1 and TOX [36]. Thus, we detected the senescent T cell phenotype and found that overexpression of p16 or p19 increased the cell size granularity (SSC), but not the cell size (FSC) (Fig. 3A). Further flow cytometric analysis showed that p16^OE^ led to increased GzmB expression (Fig. 3B), downregulation of TCR complex subunits Vα2 and Vβ5 of OT-1 cells (Fig. 3C, D). In addition, p16^OE^ reduced the expression of TCF-1, a key transcription factor associated with memory T cell formation, but did not alter the expression of PD-1, TOX, and KLRG1 (Fig. 3C, D and Supplementary Fig. S3A). p19^OE^ also did not induce a senescent T cell phenotype (Fig. 3C, D and Supplementary Fig. S3A). Additionally, the expression of SASP-related genes, including *p21*^*CIP1*^, *Il-6*, and *Cxcl1*, was not affected by high levels of p16 or p19 (Supplementary Fig. S3B). Moreover, GSEA enrichment analysis showed that overexpression of p16 or p19 alone failed to induce expression of genes related to cellular senescence (Fig. 3E, F). These observations indicate that high levels of p16 or p19 alone are insufficient to induce classical senescence in T cells.

### The upregulation of p16-induced inhibition of energy metabolism resulted in suppression of CD8^+^ T cell effector function

**Fig. 4:**
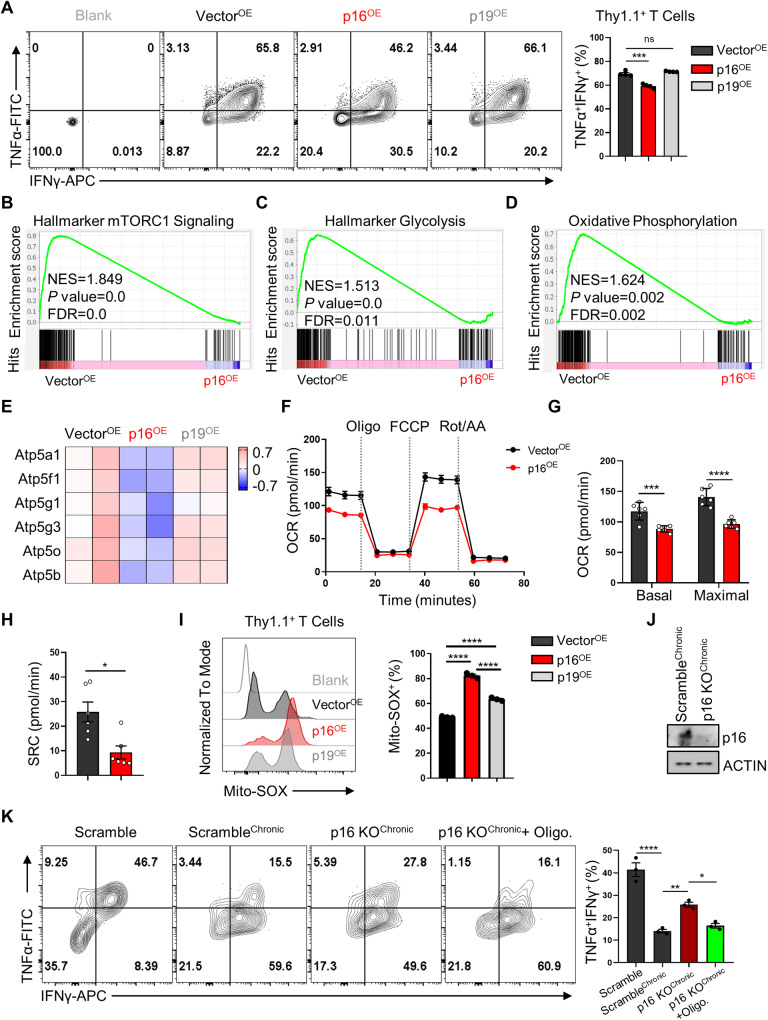
Upregulation of p16 restricted CD8^+^ T cell effector function by metabolic reprogramming. **A** Representative contour plots showing p16 or p19 overexpressing OT-1 cells producing TNF-α and IFN-γ. Right: Summary of the frequency of OT-1 cells producing cytokines. Data are shown as mean ± SEM (*n* = 3), one-way ANOVA, ***P < 0.001; ns not significant. **B–D** GSEA plot comparing Vector^OE^ with p16^OE^ CD8^+^ T cells for mTORC1, Glycolysis and Oxidative Phosphorylation associated gene sets. NES, normalized enrichment score. **E** Heatmap of selected genes encoding ATP-synthetase subunits in Vector^OE^, p16^OE^ or p19^OE^ RNA-seq. **F** The OCR of Vector^OE^ and p16^OE^ T cells was measured in real-time under basal conditions in response to the indicated inhibitors. FCCP refers to carbonyl cyanide-p-trifluoromethoxyphenylhydrazone, while ROT/AA stands for rotenone and antimycin A. Representative statistical analyses of basal OCR, maximal respiration (**G**), and SRC (**H**) are shown. Data are shown as mean ± SEM (*n* = 6), Student’s *t* test, *P < 0.05; ***P < 0.001; ***P < 0.0001. **I** Left: the mitochondrial ROS levels in OT-1 cells with indicated treatment were labeled by Mito-SOX, a representative histogram of Mito-SOX expression in indicated cells. Right: statistical analysis of percentages of Mito-SOX^+^ in these three groups. Data are shown as mean ± SEM (*n* = 3), one-way ANOVA, ****P < 0.0001. **J** CRISPR-Cas9 OT-1 cells were transfected with either Scramble or sg-RNA targeting p16. Following 24 h, T cells were re-stimulated with OVA peptide (10 nM) and maintained in culture for an additional 4 days. Western blot analysis of p16 expression in Scramble and p16 KO OT1 cells. **K** Scramble and p16 KO OT1 cells were re-stimulated with OVA peptide in the presence or absence of Oligomycin (5 nM) continuously for 4 days. Left: the representative FACS plots of TNF-α, IFN-γ production were shown. Right: summary of the proportion of TNF-α^+^ IFN-γ^+^ population. Data are shown as mean ± SEM (*n* = 3), one-way ANOVA, *P < 0.05; **P < 0.01; ***P < 0.001.

Apart from the alterations in surface markers induced by p16^OE^, our flow cytometric analysis indicated that p16^OE^ reduced the cytotoxicity of both CD8^+^ and CD4^+^ T cells, as evidenced by a decrease in the proportion of the TNF-α^+^IFN-γ^+^ population (Fig. 4A and Supplementary Fig. S4A, B). Both the capacity of cytokine production and T cell differentiation are closely linked to metabolic reprogramming [10, 37, 38]. Therefore, we analyzed whether p16^OE^ affects the metabolic features of CD8^+^ T cells. GSEA enrichment analysis showed that overexpression of p16 or p19 inhibited the mTORC1 signaling pathway (Fig. 4B and Supplementary Fig. S4C). In response to diverse stimuli, mTOR integrates multiple signal inputs to control cell anabolism, protein synthesis and cell growth or proliferation [39]. To this end, we further utilized puromycin incorporation to measure protein synthesis [40] and found that p16^OE^ indeed inhibited protein synthesis (Supplementary Fig. S4D).

Previous reports have indicated that CD8^+^ effector T cells primarily rely on aerobic glycolysis and oxidative phosphorylation (OXPHOS) to support their proliferation and effector function [40, 41]. Our GSEA analysis revealed that p16^OE^, but not p19^OE^, significantly reduced the expression of genes associated with glycolysis and OXPHOS (Fig. 4C, D and Supplementary Fig. S4E, F). Specifically, several subunits of ATP synthase, also known as complex V (cV), which couples the electron transport chain (ETC) and OXPHOS to drive ATP generation [42], were downregulated by p16 (Fig. 4E), suggesting that high levels of p16 could induce T cell metabolic inhibition. Indeed, the Seahorse analysis revealed that p16^OE^ T cells exhibited reduced basal and maximum oxygen consumption rate (OCR) and spare respiratory capacity (SRC) (Fig. 4F–H). Considering the crucial role of mitochondrial metabolism in T cell survival and differentiation [44], we investigated the quantity and quality of mitochondria in the three groups. We measured mitochondrial mass, membrane potential, and reactive oxygen species (ROS) levels using MitoTracker green (MTG), tetramethyl rhodamine ethyl ester (TMRE), and MitoSOX, respectively. Both p16 and p19 overexpression led to increased mitoROS generation (Fig. 4I), but did not alter mitochondrial quantity (Supplementary Fig. S4G). Notably, p16^OE^ T cells exhibited a higher proportion of high-membrane potential population (TMRE^high^) compared to Vector^OE^ or p19^OE^ cells (Supplementary Fig. S4G), consistent with the inhibition of ATP synthase function. Moreover, to confirm the role of mitochondrial metabolic damage in contributing to T cell functional suppression, we generated p16 knockout T cells using the CRISPR-Cas9 method (Fig. 4J). We observed that deletion of p16 partially rescued the population of TNF-α^+^IFN-γ^+^ cells under prolonged antigen exposure, which had been suppressed by the mitochondrial ATP synthase inhibitor, Oligomycin (Fig. 4K). In summary, these results suggest that upregulation of p16 impairs mitochondrial metabolism, leading to suppression of effector function in CD8^+^ T cells.

### The high expression of p16 depends on CDK4/6 inhibition to promote CD8^+^ T cell exhaustion

**Fig. 5:**
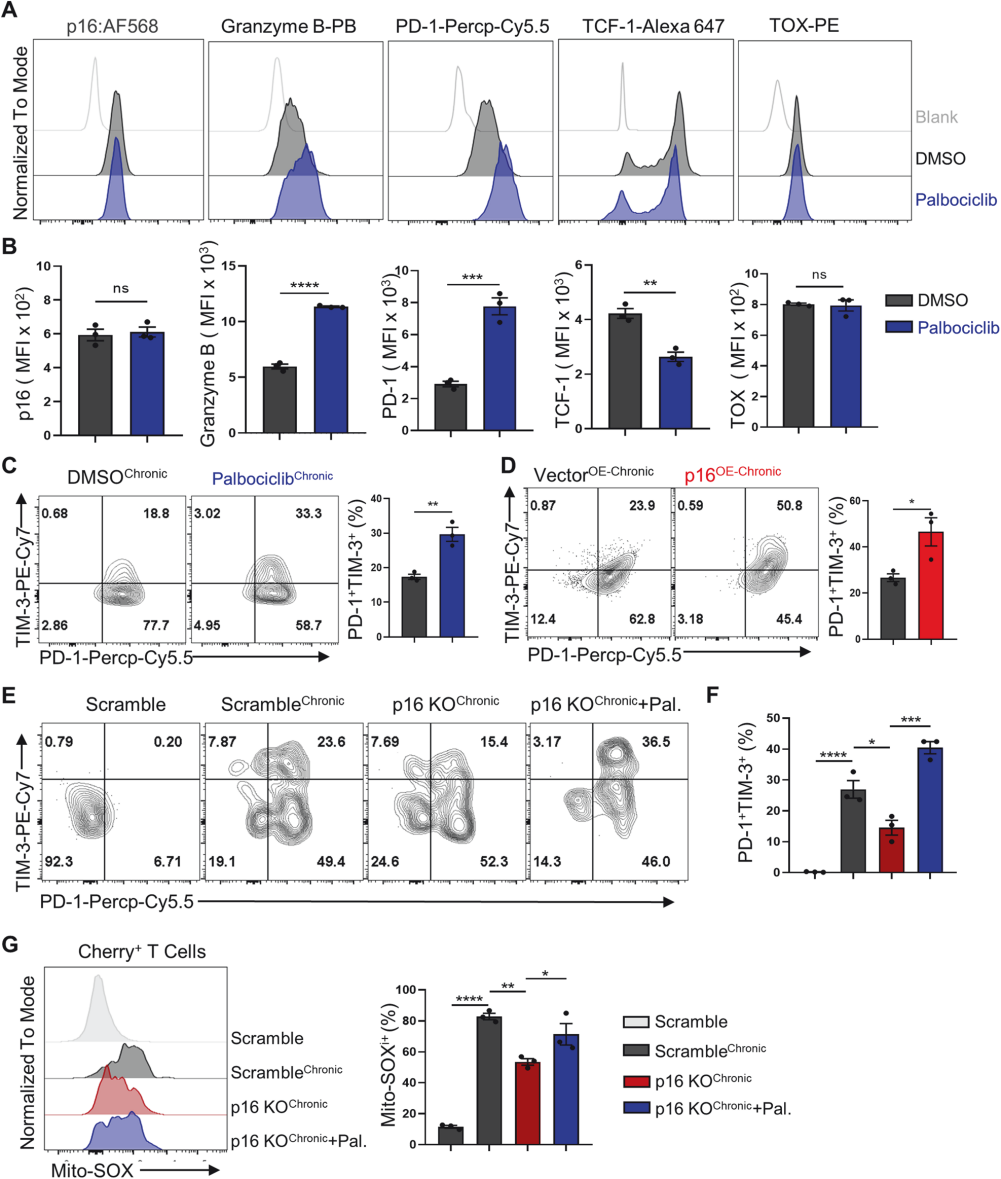
High expression of p16-mediated exhaustion of CD8^+^ T cell in vitro was dependent on CDK4/6 inhibition. **A**, **B** Flow cytometry analysis plots of OT-1 cells treated with DMSO or Palbociclib (500 nM). (up). MFI of indicated proteins (p16, Granzyme B, PD-1, TCF-1, TOX) in various OT-1 cells were shown (down). Data are shown as mean ± SEM (*n* = 3), Student’s *t* test, **P *<* 0.01; ***P < 0.001; ****P < 0.0001; ns not significant. **C** Activated OT-1 cells were re-stimulated continuously with OVA peptides in the presence of either DMSO or Palbociclib, followed by flow cytometry analysis of PD-1 and TIM-3 expression (left panel). The percentages of PD-1^+^TIM-3^+^ cells were compared (right panel). Data are shown as mean ± SEM (*n* = 3), Student’s *t* test, **P *<* 0.01. **D** OT-1 cells overexpressing control vector or p16 were re-stimulated with continuous OVA peptides for 4 days (left), and then the percentages of PD-1^+^TIM-3^+^ were compared (right). Data are shown as mean ± SEM (*n* = 3), Student’s *t* test, *P *<* 0.05. **E**, **F** Scramble and p16 KO OT1 cells were re-stimulated with OVA peptide and treated with or without Palbociclib continuously for 4 days. Flow cytometry analysis of PD-1 and TIM-3 expression in Cherry^+^ cells were shown on the left. Percentages of PD-1^+^TIM-3^+^ cells were compared on the right. **G** Mitochondrial redox levels in OT-1 cells with indicated treatments were marked by Mito-SOX (left), and a summary of the percentages of Mito-SOX^+^ cells in the four groups was compared on the right. Data are shown as mean ± SEM (*n* = 3), one-way ANOVA, *P *<* 0.05; **P *<* 0.01; ***P *<* 0.001; ****P *<* 0.0001. Data are shown as mean ± SEM (*n* = 3), one-way ANOVA, *P *<* 0.05; ***P *<* 0.001.

The p16-induced cell cycle arrest primarily occurs through CDK4/6 inhibition. To investigate whether the phenotype caused by p16^OE^ in CD8^+^ T cells was due to CDK4/6 inhibition, we compared the phenotype of p16^OE^to CDK4/6 inhibitor treatment. RNA sequencing results revealed that p16^OE^ decreased the expression of E2F target genes in CD8^+^ T cells (Supplementary Fig. S5A), suggesting that CDK4/6 inhibition contributed to the phenotype induced by p16^OE^. We further treated OT-1 cells with CDK4/6 inhibitor palbociclib. While palbociclib treatment did not affect intrinsic p16 expression, similar to p16^OE^, it blocked cell cycle progression of CD8^+^ T cells in vitro (Supplementary Fig. S5B), increased GzmB expression, and decreased TCF-1 expression (Fig. 5A, B). Furthermore, both the mitochondria membrane potential and mitoROS were increased under palbociclib treatment (Supplementary Fig. S5C, D). However, we further demonstrated that palbociclib upregulated the proportion of the TNF-α^+^IFN-γ^+^ population (Supplementary Fig. S5E), which contradicted the downregulation of effector function caused by p16^OE^, suggesting that palbociclib might have other unknown effects on T cell function.

To further detect whether the upregulation of p16 contributes to T cell dysfunction in a CDK4/6-dependent manner, we employed a persistent TCR stimulation model, as previously described, to induce T cell exhaustion in vitro [17] (Supplementary Fig. S5F). We observed that both p16^OE^ and palbociclib treatment increased the proportion of PD-1^+^TIM-3^+^ terminally exhausted cells (Fig. 5C, D). Consistently, knockout of p16 partially mitigated T cell terminal exhaustion and reduced mitoROS production under prolonged antigen exposure, while treatment with palbociclib eliminated this advantage (Fig. 5E–G). These results suggest that the dysfunction of CD8^+^ T cells induced by p16 upregulation primarily depends on its inhibition of CDK4/6.

### The upregulation of p16 promoted terminal exhaustion of tumor-infiltrating CD8^+^ T cells

**Fig. 6:**
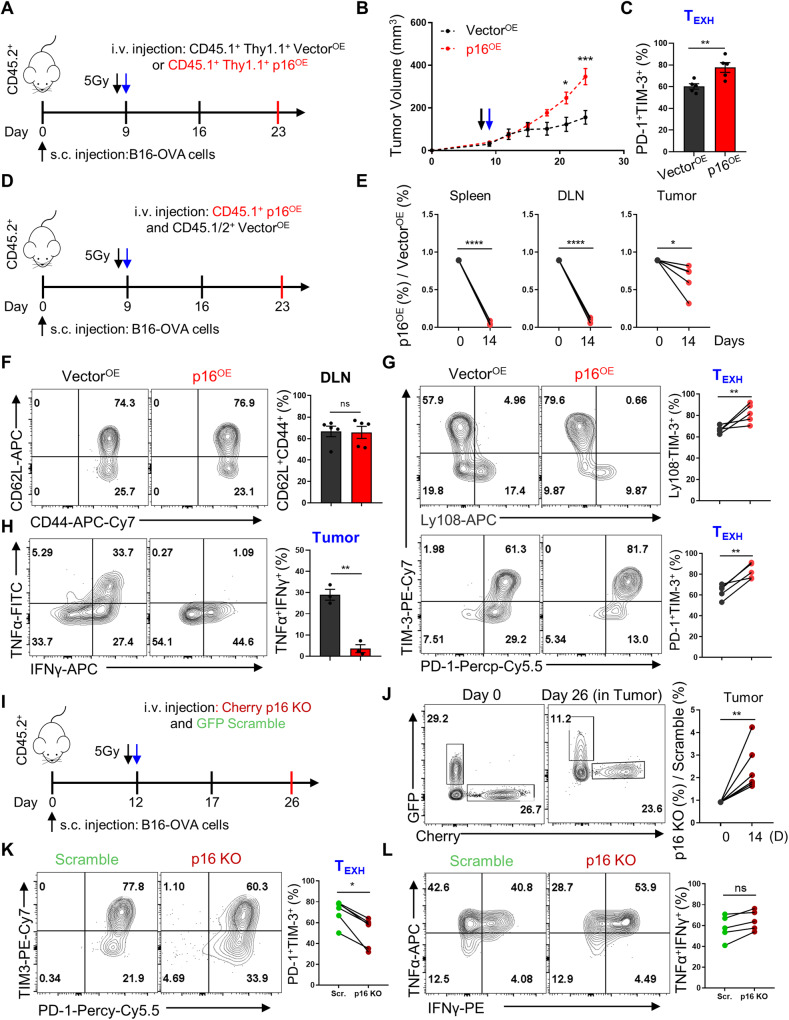
High expression of p16 restricted T cell persistence and promoted exhaustion differentiation of TILs in vivo. **A** Schematic representation illustrating T cell adoptive transfer in the tumor model. **B** Tumor growth curves of B16-OVA tumor models pre- and post-transfer of Vector^OE^ and p16^OE^ OT-1 cells. Data are shown as mean ± SEM (*n* = 5), two-way ANOVA analysis, *P < 0.05; ****P < 0.0001. **C** Values indicate the frequency of PD-1^+^TIM-3^+^ in Vector^OE^ and p16^OE^ OT-1 TILs. Data are shown as mean ± SEM (*n* = 5), Student’s *t* test, **P *<* 0.01. **D** Diagram illustrating co-adoptive transfer of T cells in the tumor model. **E** Kinetics of Vector^OE^ and p16^OE^ OT-1 cells in the spleen, DLN, and tumor of recipient mice at 14 days post-i.v., respectively. **F** Left: CD44 and CD62L expression in Vector^OE^ or p16^OE^ OT-1 cells from draining lymph nodes (DLN) for each individual. Right: summary of the CD62L^+^CD44^+^ frequencies of indicted OT-1 cells for multiple. **G** Left: Representative FACS plots showing Ly108, PD-1, and TIM-3 expression in indicated OT-1 TILs. Right: values indicate the T_EXH_ frequencies, identified by Ly108^-^TIM-3^+^ and PD-1^+^TIM-3^+^ populations in OT-1 TILs. Data are shown as mean ± SEM (*n* = 5), Student’s *t* test, **P *<* 0.01; ns not significant. **H** Left: Representative contour plots of indicated OT-1 TILs producing cytokines TNF-α and IFN-γ after stimulation with PMA and Ionomycin. Right: summary of the proportion of indicated Thy1.1^+^ OT-1 TILs producing cytokines. Data are shown as mean ± SEM (*n* = 3), Student’s *t* test, **P *<* 0.01. **I** Schematic depicting the study design. **J** Representative FACS plots of Scramble (GFP) and p16 KO (Cherry) OT1 cells within total tumor-infiltrating CD8^+^ T cells of recipient mice at 28 days and before transfer (left). Kinetics of the co-transferred OT1 cells within tumor at 14 days post transfer (right). **K** Left: Representative FACS plots of PD-1, and TIM-3 expression in indicated OT-1 TILs. Right: Summary of the T_EXH_ frequencies of PD-1^+^TIM-3^+^ in among the indicated OT-1 TILs. **L** Left: Representative FACS plots of TNF-α, and IFN-γ expression in indicated OT-1 TILs. Right: values indicate the frequencies of TNF-α^+^IFN-γ^+^ populations in OT-1 TILs. Data are shown as mean ± SEM (*n* = 5), Student’s *t* test, *P < 0.05; **P < 0.01; ns not significant.

Based on previous observations, we performed an adoptive cell transfer (ACT) experiment to validate the role of high-level p16 expression in T cell exhaustion (Fig. 6A). As expected, the p16^OE^ significantly compromised the anti-tumor efficacy of adoptively transferred T cells (Fig. 6B). The proportion of adoptively transferred p16^OE^ T cells was significantly lower than Vector^OE^ T cells in both the spleen and tumor tissues (see Supplementary Fig. S6A, B). Moreover, p16^OE^ T cells exhibited a higher proportion of terminally exhausted population (PD-1^+^TIM-3^+^) compared to the Vector^OE^ group (Fig. 6C and Supplementary Fig. S6C). To avoid any effects resulting from varied host microenvironments, we next utilized a co-transfer strategy, whereby a 1:1 ratio of Vector^OE^ and p16^OE^ OT-1 cells were introduced into the same B16-OVA-bearing mice (Fig. 6D). After two weeks, the proportion of p16^OE^ OT-1 cells was significantly lower in the spleen, DLN, and the tumor tissues compared to Vector^OE^ (Fig. 6E). Since it has been reported that tumor-specific CD8^+^ T cells treated with CDK4/6 inhibitors promote T-cell persistence and the formation of immunological memory. Specifically, the inhibition of CDK4/6 upregulates MXD4, a negative regulator of MYC, thereby prompting the differentiation of CD8^+^ T cells into a central memory state [16]. In our study, we analyzed the central memory population (CD62L^+^CD44^+^) in lymph node and found a similar proportion of CD62L^+^CD44^+^ cells between the p16^OE^ and Vector^OE^ groups (Fig. 6F). This indicates that high levels of p16-induced cell cycle arrest do not induce the differentiation of CD8^+^ T cells into central memory cells in vivo. Instead, further flow cytometry analysis revealed an increased proportion of Ly108^-^TIM-3^+^ and PD-1^+^TIM-3^+^ terminally exhausted populations in p16^OE^ group (Fig. 6G). Consistently, tumor-infiltrating CD8^+^ T cells with p16 overexpression demonstrated reduced frequency of TNF-α^+^IFN-γ^+^ population (Fig. 6H), indicating that p16^OE^ impaired CD8^+^ T cell effector function at the tumor site.

Furthermore, we validated the impact of p16 knockout on T cell exhaustion in vivo using a co-transfer strategy. In this approach, a 1:1 ratio of scramble (GFP) and p16 knockout (Cherry) CRISPR-Cas9 OT-1 cells were simultaneously transferred into B16-OVA bearing mice (Fig. 6I). Consistent with previous findings, p16 knockout OT-1 cells exhibited a distinct survival advantage in the spleen, DLN, and tumor tissues (Fig. 6J and Supplementary Fig. S6D). Correspondingly, the population of terminally exhausted T cells was diminished in the adoptively transferred T cells lacking p16 (Fig. 6K). Although there was a trend towards elevated TNF-α^+^IFN-γ^+^ population in p16 knockout cells, the difference was not statistically significant (Fig. 6L). In summary, increased levels of p16 promoted exhaustion of tumor-infiltrating CD8^+^ T cells, leading to a reduction in the effectiveness of ACT immunotherapy, while p16 knockout blocked T cell terminal exhaustion and facilitated its persistence in vivo.

## Discussion

The exhaustion and limited persistence of tumor antigen-specific T cells represent significant challenges for CD8^+^ T cell immunotherapy [3]. However, the link between cell cycle regulation and exhausted T cell differentiation remains unclear. In this study, we demonstrated that the expression of the cell cycle regulator p16 in CD8^+^ T cells was rapidly increased following TCR stimulation. Elevated p16 levels not only inhibited cell proliferation but also triggered T cell apoptosis independent of DNA damage. Consequently, the lower expression of p16 in terminally exhausted T cells compared to the precursor subset in vivo may be attributed to the higher susceptibility of T cells with elevated p16 expression to death and clearance. However, the maintenance of the remaining terminally exhausted T cells appears unaffected by p16 expression. Additionally, increased levels of p16 led to inhibition of mitochondrial metabolism, contributing to the differentiation of T cells towards terminal exhaustion rather than classical senescence. Further ACT experiments revealed that increased p16 expression in T cells significantly impaired their ability to mount an effective anti-tumor immune response by diminishing T cell persistence and promoting terminal exhaustion of tumor-infiltrating T cells in vivo. Conversely, p16 knockout resulted in reduced T cell terminal exhaustion both in vitro and in vivo. In summary, our data supported that p16 is upregulated by TCR stimulation and serves as a critical regulator that facilitates the exhaustion of tumor-infiltrated T cells.

The tumor suppressors p16 and p19 (known as p14 in humans) are two isoforms encoded by the Cdkn2a gene, yet they exert distinct effects on cell growth and tumorigenesis [43–45]. While p16 inhibits CDK4/6 and induces oncogene-induced senescence to suppress tumors [46, 47], p19 functions to inhibit MDM2-mediated degradation of p53 [48, 49]. We compared the phenotypes mediated by p19^OE^ to those of p16^OE^, as both were increased in exhausted T cells (Supplementary Fig. S1B, D). Interestingly, p16 and p19 overexpression shared similar characteristics, such as blocking proliferation, increasing apoptosis, and inhibiting mTOR signaling-associated gene expression. However, only p16^OE^ inhibited the expression of glycolysis and OXPHOS-associated genes (Fig. 4C, D and Supplementary Fig. S4E, F). Furthermore, p19^OE^ had no impact on mitochondria membrane potential and T cell effector function (Fig. 4A and Supplementary Fig. S4G), suggesting that p16 and p19 may play differential roles during T cell exhaustion differentiation.

In senescent cells, the accumulation of DNA damage and reactive oxygen species (ROS) activates the p16–RB pathway, which inhibits the transcription of cell-cycle genes [11]. Consequently, p16 is often considered an aging marker. Moreover, CDK4/6 inhibitors, which mimic the function of p16, have been demonstrated to induce senescence in various cancer cells [50–53], reducing the proliferative capacity of activated T cells while preserving their cytotoxic efficacy [54]. However, it is still uncertain whether cell cycle arrest alone is enough to induce T cell senescence. Given that cellular senescence is usually initiated by DNA damage, the influence of p16-mediated cell cycle arrest on T cell differentiation might be masked by DNA damage. To address this, we employed a model of persistent antigenic stimulation to explore the expression and function of p16 in T cell exhaustion, mimicking sustained stimulation by tumor antigens rather than the chronic replicative stress seen in long-term culture models. Of note, p16^OE^ alleviated replication-induced DNA damage (Fig. 2D). Our findings showed that antigen stimulation promptly increased p16 expression, resulting in T cell death or differentiation into a terminally exhausted state both in vitro and in vivo, rather than classical senescence. Therefore, those discrepancies among various investigations of p16 effects on T cell differentiation may due to T cell heterogeneity, and distinct experimental conditions.

Additionally, senescent cells exhibit notable resistance to apoptosis [55, 56]. Previous research has linked this resistance to impaired p53 signaling, heightened activity of the NF-κB-IAP/JNK axis, and alterations in epigenetic regulation [56, 57]. In this study, we specifically modulated the expression of p16 or p19 without introducing factors such as p53 deficiency or DNA damage during cellular senescence. Our results demonstrated that the expression of p16 or p19 alone was not sufficient to induce T-cell senescence, suggesting that T cell senescence is driven by a combination of multiple factors, including not only cell cycle blockade but also DNA damage, epigenetic alterations, mitochondrial damage, and activation of inflammatory signaling pathways. Further research is needed to explore these mechanisms.

In summary, our findings suggest that prolonged exposure to antigens triggers increased p16 expression, which inhibits T cell proliferation and promotes terminal T cell exhaustion. Given the significance of tumor antigen-specific T cells in the draining lymph nodes for the response to anti-PD-1/PD-L1 therapy [58], our co-transferred ACT experiment revealed a limited presence of p16^OE^ CD8^+^ T cells in the spleen and draining lymph nodes. Those observations suggest that elevated p16 levels in T cells might compromise the effectiveness of anti-tumor therapy. Thus, targeting the CDK4/6 pathway in tumor-infiltrated T cells holds promise for enhancing the efficacy of anti-tumor immunotherapy.

## Materials and methods

### Cell lines

B16 (ATCC, CRL-6475) and B16-OVA cells were cultured in RPMI medium (Gibco, 11875-093) supplemented with 10% FBS (Cytia, SH30396) and Pen/Strep (Hyclone, SV30010) at 37 °C with 5% CO_2_. 293FT cells (ATCC, CRL-3249) and MC38 cells (ATCC, CRL-2640) were grown in DMEM medium (Gibco, C11995500CP) supplemented with 10% FBS and penicillin–streptomycin and grown in the same conditions as noted above. Regular checks were performed to ensure there was no mycoplasma contamination in the cell lines.

### Mice

Animal protocols were reviewed and approved by the Institutional Animal Care and Use Committee (IACUC) of Suzhou Institute of System Medicine (ISM-IACUC-0018 and ISM-IACUC-0055). The wild-type CD45.1^+^ or CD45.1^+^CD45.2^+^ OT-1 TCR transgenic male and female mice, as well as CRISPR-Cas9 CD45.1^+^ OT-1 TCR transgenic female mice on a C57BL/6N background were housed. CD45.2^+^ female C57BL/6N mice (6–8 weeks old, WT) were purchased from Vital River Co, Ltd (Beijing, China) as recipients. For each independent in vivo experiment, sex-matched CD45.1^+^ and CD45.2^+^ mice aged 6 to 12 weeks were utilized. All of the mice were bred and maintained under specific pathogen-free conditions in the animal facility of Suzhou Institute of Systems Medicine (Suzhou, China). All of the experiments were conducted in accordance with the relevant regulations of the committee.

### DNA cloning

To overexpress p16 or p19 in OT-1 cells, mouse p16 or p19 sequences were cloned into the XhoI (NEB, R0146S) and HindIII (NEB, R3104V) sites of the pMSGV-Thy1.1 vector. The primer sequences used for cloning are as follows: p16 (froward 5′–3′ CCGCTCGAGATGGAGTCCGCTGCAGACAGAC; reverse: 5′–3′ CCAAGCTTGGGCTCTGCTCTTGGGATTGGCC); p19 (froward 5′–3′ CCGCTCGAGATGGGTCGCAGGTTCTTGGTCAC; reverse: 5′–3′ CCAAGCTTGGTGCCCGTCGGTCTGGGCGACG).

### *p16*^*INK4a*^ knockout

The CRISPR/Cas9 system was used to generate *p16*^*INK4a*^ knockout T cells. The scramble sg-RNA (TGGATTTAGGGCTGCCTTCC) and mouse p16-targeting sgRNA (GTGCGATATTTGCGTTCCGC) were inserted into pSL21-GFP or pSL21-mCherry retroviral plasmid using T4 DNA Ligase (Vazyme, C301-01). The packaged retroviruses were then used to infect Cas9-OT1 cells.

### Retrovirus packaging and transfection

The helper plasmid pCL-Eco and the specified pMSGV or pSL21-mCherry retroviral plasmids were co-transfected into 293FT cells using Trans-IT 293 transfection reagent (Mirusbio, MIR2700). The resulting supernatants containing viruses were then mixed with anti-CD3/CD28 activated OT-1 cells at a multiplicity of infection (MOI) of 5:1, along with polybrene (1 μg/mL) (Santa Cruz, SC-134220). Subsequently, the mixtures were added to a 24-well plate precoated with RetroNectin (0.1 mg/mL) (TaKaRa, T100B). After centrifugation at 2000×*g* for 90 min, the cells were cultured at 37 °C under 5% CO2 for 24 h. Infected cells were subsequently detected using flow cytometry.

### CD8^+^ T cell activation

The culture RPMI medium contains 10% FBS, Hepes (10 mM, Bioind, 03-025-1B), Sodium Pyruvate (1 mM, Gibco, 11360-070), penicillin–streptomycin, l-Glutamine (2 mM, Gibco, 25030081), MEM Non-Essential Amino Acids (1×, Thermo, 11140050) and β-mercaptoethanol (50 μM, Sigma, B6891). Then 2 × 10^6^ splenocytes obtained from OT-1 mice were plated into each well of a 24-well plate containing 2 mL of medium supplemented with OVA peptide (1 μg/mL, Abcepta Biotech, SP1050a) and IL-2 (10 ng/mL, eBioscience, 17-7029-82). After 3 days, dead cells were eliminated using Ficoll-Paque (Sigma, GE17-1440-03), and the viable CD8^+^ T cells were further cultured in medium supplemented with IL-2 and IL-7 (both at 10 ng/mL, Novoprotein, CX47). For retroviral infection expriments, naive CD8^+^ T cells were isolated from the spleens of OT-1 mice (aged 6–8 weeks) using a commercial kit (Biolegend, 480031). Then, 1 × 10^6^ cells were seeded into each well of a 48-well plate containing 1 mL of RPMI medium supplemented with IL-2, which had been precoated with αCD3/CD28 antibodies (Invitrogen, 16-0031-86, 16-0281-85). After 2 days, the activated CD8^+^ T cells were infected with indicated retrovirus.

### Adoptive T cell therapy in tumor-bearing mice

Tumors were implanted in C57BL/6 female mice by injecting 5 × 10^5^ B16-OVA tumor cells per mouse subcutaneously (s.c.) into the right flank at day 0. Once the tumors reached approximately 4–5 mm in diameter, equal numbers of the specified cells (1 × 10^6^ per mouse) from female OT-1 mice (6–8 weeks old) overexpressing Vector or p16 were intravenously (i.v.) transferred into recipient mice on the day following irradiation (5 Gy). Tumor dimensions were assessed every 3 days using a digital Vernier caliper, and tumor volume was determined using the formula V = (L × W^2^)/2, where V represents tumor volume, L is the length of the tumor (longer diameter), and W is the width of the tumor (shorter diameter).

### Co-transfer strategy

Naive CD8^+^ T cells were isolated from the spleens of wild-type CD45.1^+^ or CD45.1^+^CD45.2^+^ OT-1 mice, followed by infection with Vector or p16 viruses, respectively. After irradiation, CD45.1^+^CD45.2^+^ Vector^OE^ and CD45.1^+^p16^OE^ OT-1 cells were mixed at a 1:1 ratio, a total of 2 × 10^6^ cells and transferred into B16-OVA tumor-bearing mice as indicated. For analysis of p16 knockout during T cell exhaustion in vivo, the naive CD8^+^ T cells were isolated from Cas9-OT1 mice spleen, then infected cells with Scramble-GFP or p16 sg-RNA-Cherry viruses, respectively. After irradiation, GFP^+^ and Cherry^+^ OT-1 cells were mixed at a 1:1 ratio, a total of 2 × 10^6^ cells were transferred into B16-OVA tumor-bearing mice as indicated.

### Flow cytometry

Cells were subjected to fluorescent antibody staining and subsequently analyzed via flow cytometry. For cell surface staining, all antibodies were diluted at a ratio of 1:400 with FACS buffer (PBS containing 2% FBS) and incubated for 20 min on ice. The antibodies used included Brilliant Violet711 anti-CD8a (Biolegend, 100748), Alexa Fluor700 anti-CD90.1 (Biolegend, 202536), APC anti-TCR Vα2 (Biolegend, 127810), FITC anti-TCR Vβ5 (BD Biosciences, 557004), PerCP-eFluor710 anti-PD-1 (Invitrogen, 46-9981-82), PE-Cy7 anti-TIM-3 (Invitrogen, 25-5870-82), APC anti-CD62L (Biolegend, 104412), PE anti-CD45.1 (Biolegend, 110708), and FITC anti-CD45.2 (Invitrogen, 11-0454).

For intracellular cytokine staining, cells were incubated with PMA (10 ng/mL) (Sigma-Aldrich, P1585), ionomycin (50 μg/mL) (Fcmacs Biotech, FMS-FZ208), Brefeldin A (Invitrogen, 00-4506-51) and Monensin (Invitrogen, 00-4505-51) for 4 h at 37 °C. Then, cells were pre­stained with Live/Dead Fixable Dead Cell Stain Kit (Invitrogen, 65-0866-18), then the pre­stained cells were fixed with Fixation Buffer (Biolegend, 420801) for 20 min on ice, permeabilized with Permeabilization Buffer (Biolegend, 421002) and stained with indicated antibodies, which including FITC-anti-TNF-α (Biolegend, 506304), APC-anti-IFN-γ (Invitrogen, 17-7311-82).

For intracellular transcription factor staining, cells were first pre-stained with Live/Dead and fluorescently conjugated antibodies in FACS buffer to detect surface markers. Then, cells were fixed and permeabilized using Foxp3/Transcription Factor Staining Buffer (Invitrogen, 00-5123-43 and 00-5223-56) and Permeabilization Buffer (Invitrogen, 00-8333-56), followed by staining with antibodies. This included Alexa Fluor647-anti-TCF-1 (CST, 6709S), PE-TOX (Invitrogen, 12-6502-82), primary antibodies against p16 (Abcam, ab211542) and Phospho-Histone H2A.X (CST, 9718), and Goat anti-Rabbit Alexa Fluor568 secondary antibody (eBioscience, A-11036). All samples were resuspended in FACS buffer and analyzed using an LSR Fortessa flow cytometer (Becton-Dickinson, San Jose, CA) with FlowJo software.

### OCR measurement

The OCR was assessed using a Seahorse instrument (XF24, Agilent). Briefly, the indicated T cells were treated with non-buffered XF medium (Agilent, 103576-100), which containing 10 mM glucose, 1 mM sodium pyruvate, and 2 mM glutamine. Subsequently, the T cells were seeded at 12 × 10^4^ cells per well in a XF96 cell culture microplate and incubated in a non-CO_2_ incubator for 1 h at 37 °C. Oxygen consumption was then analyzed under basal conditions and in response to 1.25 μM oligomycin, 1.5 μM FCCP, 0.5 μM rotenone, and 0.5 μM antimycin A (Agilent, 103015-100). The SRC was determined by subtracting the basal OCR from the maximum OCR.

### Western blot

The indicated cells were harvested and washed with cold PBS, followed by extraction of total protein using 1% SDS on ice and heating in a water bath at 100 °C for 15 min. The protein samples were then separated by SDS–PAGE gels and transferred onto Nitrocellulose membranes (Bio-Rad). After blocking with 5% nonfat milk in PBST, the membranes were incubated with primary antibodies against anti-BCL-XL (CST, 2764) or anti-BAX (CST, 14796) overnight at 4 °C, followed by goat anti-rabbit HRP-coupled secondary antibody (Absin, abs20040ss) for 2 h at room temperature. The HRP signal was visualized using electrochemiluminescence (ChemeMINI610) and captured using Sage Capture (v2.19.12). Data analysis was conducted using ImageJ (v1.8.0) software.

### Quantitative PCR

For the analysis of in vitro antigen-stimulated OT-1 cells, dead OT-1 cells were eliminated using Ficoll-Paque. The total RNA of viable indicated cells was then extracted using the RNeasy Mini Kit (Qiagen, 74104) and subjected to reverse transcription using the PrimeScript RT Master Mix Kit (TaKaRa, RR036B). The reactions were conducted on a real-time PCR system (Roche LC480) using PerfectStart Green qPCR SuperMix (TransGen Biotech, AQ601-04). The primer sequences were as follows: *p16*^*INK4A*^ (froward 5′–3′: CCCAACGCCCCGAACT; reverse: 5′–3′ GCAGAAGAGCTGCTACGTGAA), *p19*^*ARF*^ (froward 5′–3′: CGCAGGTTCTTGGTCACTGT; reverse: 5′–3′ TGTTCACGAAAGCCAGAGCG), *p21*^*CIP1*^ (froward 5′–3′: GGCAGACCAGCCTGACAGAT; reverse: 5′–3′ TCAGGGTTTTCTCTTGCAGAAG), Il-6 (froward 5′–3′: CACGGCCTTCCCTACTTCAC; reverse: 5′–3′ GGTCTGTTGGGAGTGGTATC), *Cxcl1* (froward 5′–3′: TCCAGAGCTTGAAGGTGTTGCC; reverse: 5′–3′ AACCAAGGGAGCTTCAGGGTCA), *Pdcd1* (froward 5′–3′: ACCCTGGTCATTCACTTGGG; reverse: 5′–3′ CATTTGCTCCCTCTGACACTG).

### RNA sequencing

Vector^OE^, p16^OE^ and p19^OE^ CD8^+^ T cells were labeled with PE-anti-Thy1.1 antibody and sorted using flow cytometry. Subsequently, RNA was extracted from the indicated T cells using TRIzol. The integrity of the RNA was assessed using the Agilent 2100 Bioanalyzer (Agilent). Library preparation was performed using the TruSeq RNA sample prep kit (FC­122­1001, Illumina). The libraries were then subjected to sequencing on an Illumina NovaSeq 6000 platform, generating approximately 40 million paired-end reads (Novogene). RNA sequencing was performed, and the data were analyzed by the bioinformatics core at the Suzhou Institute of Systems Medicine. Source data have been made publicly available on the Figshare website with the following DOI: 10.6084/m9.figshare.25533121. The expression heat maps were generated with the R package ‘heatmap’ (v1.0.12). GSEA v.4.0 was used for GSEA analysis (Gene-set enrichment analysis).

### Quantification and statistical analysis

Data were analyzed from a minimum of three independent experiments and are presented as the mean ± SEM. The sample size was not predetermined using statistical methods but was based on previous experimental observations. Mice were randomly assigned to experimental groups, and animal experiments were conducted without blinding. Statistical comparisons between two groups were performed using Student’s two-tailed *t* test, while comparisons among three groups were analyzed using one-way ANOVA. Tumor growth curve comparisons were analyzed by two-way ANOVA. Variance was similar between the groups undergoing statistical comparisons. *P* values < 0.05 (*P < 0.05; **P < 0.01; ***P < 0.001, ****P < 0.0001) were considered significant. All analyses were conducted using GraphPad Prism 8 software (GraphPad, San Diego, USA).

### Supplementary information

Supplemental Material

Original Western blot images

## Data Availability

This paper does not report the original code. Any additional information required to reanalyze the data reported in this paper is available from the lead contact upon request.
