# Supplementary material for: The cell cycle regulator p16 promotes tumor infiltrated CD8+ T cell exhaustion and apoptosis
Source: Cell Death Dis. 2024 May 15;15(5):339. doi: 10.1038/s41419-024-06721-7 (PMC11096187; doi:10.1038/s41419-024-06721-7)
Supplement: Supplementary file 1 — Supplemental Material [file 41419_2024_6721_MOESM1_ESM.docx]

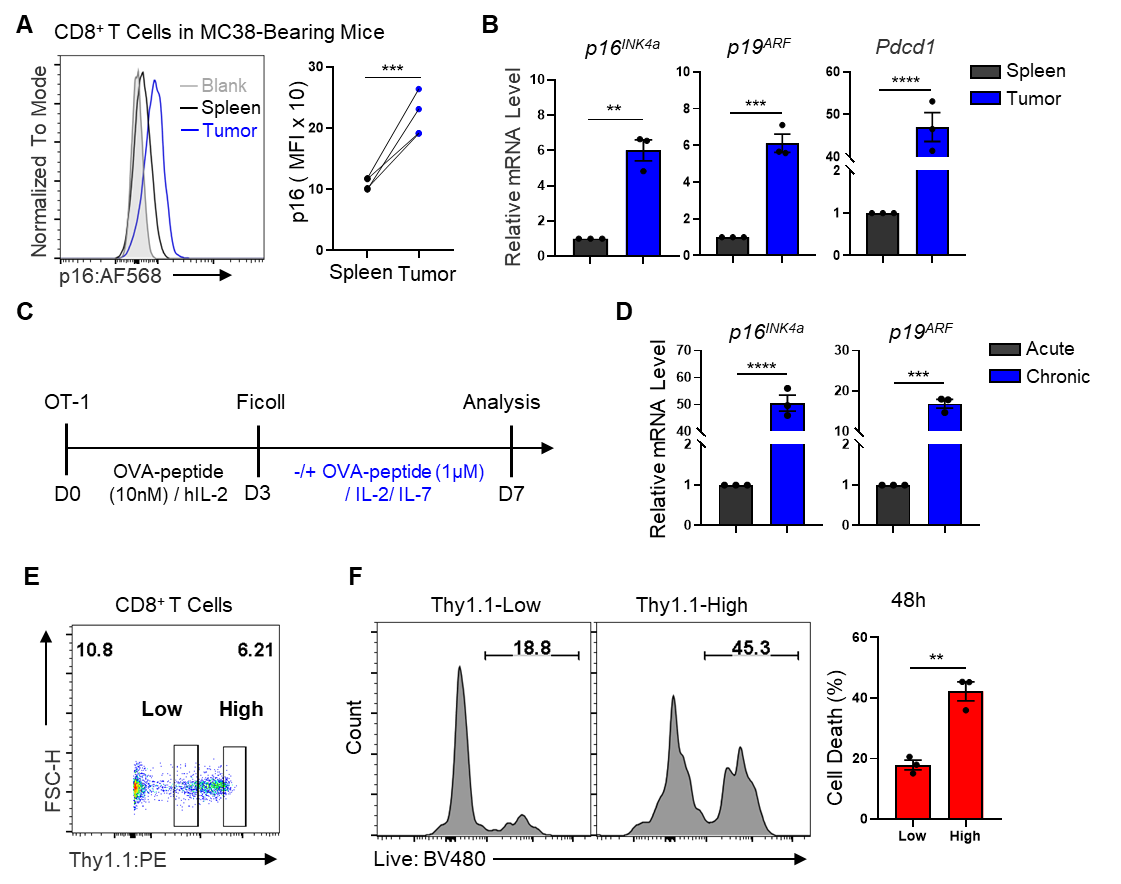


**Figure S1. Expression of p16 and p19 in OT-1 T cells under continuously stimulated by antigen**

(**A**) Left: representative histogram of p16 expression in the spleen or MC38 tumor-infiltrated OT-1 T cells. Right: Statistical analysis of p16 MFI (mean fluorescence intensity) in indicated cells. Data are shown as Mean (n=4), Student’s t test, ****: p＜0.001*. (**B**) qPCR experiment showed the relative mRNA levels of *p16^INK4a^*, *p19^ARF^*, and *Pdcd1* in the spleen or tumor-infiltrated OT-1 T cells. Mean ± SEM (n=3), Student’s t test, ***: p＜0.01; ***: p＜0.001; ****: p < 0.0001*.(**C**) Schematic representation of OT-1 cells under continuously stimulated with antigen *in vitro*. (**D**) Relative mRNA levels of *p16^INK4a^* and *p19^ARF^* in the OT-1 cells treated with continuous antigen stimulation or not by qPCR experiments. Mean ± SEM (n=3), Student’s t test, ****: p＜0.001; ****: p < 0.0001*. (**E**) Representative chart of flow sorting. (**F**) Left: representative histogram of cell death in p16 low or high expressing cells. Right: Statistical analysis of indicated cell death after sorting 48h. Mean ± SEM (n=3), Student’s t test, ***: p＜0.01.*


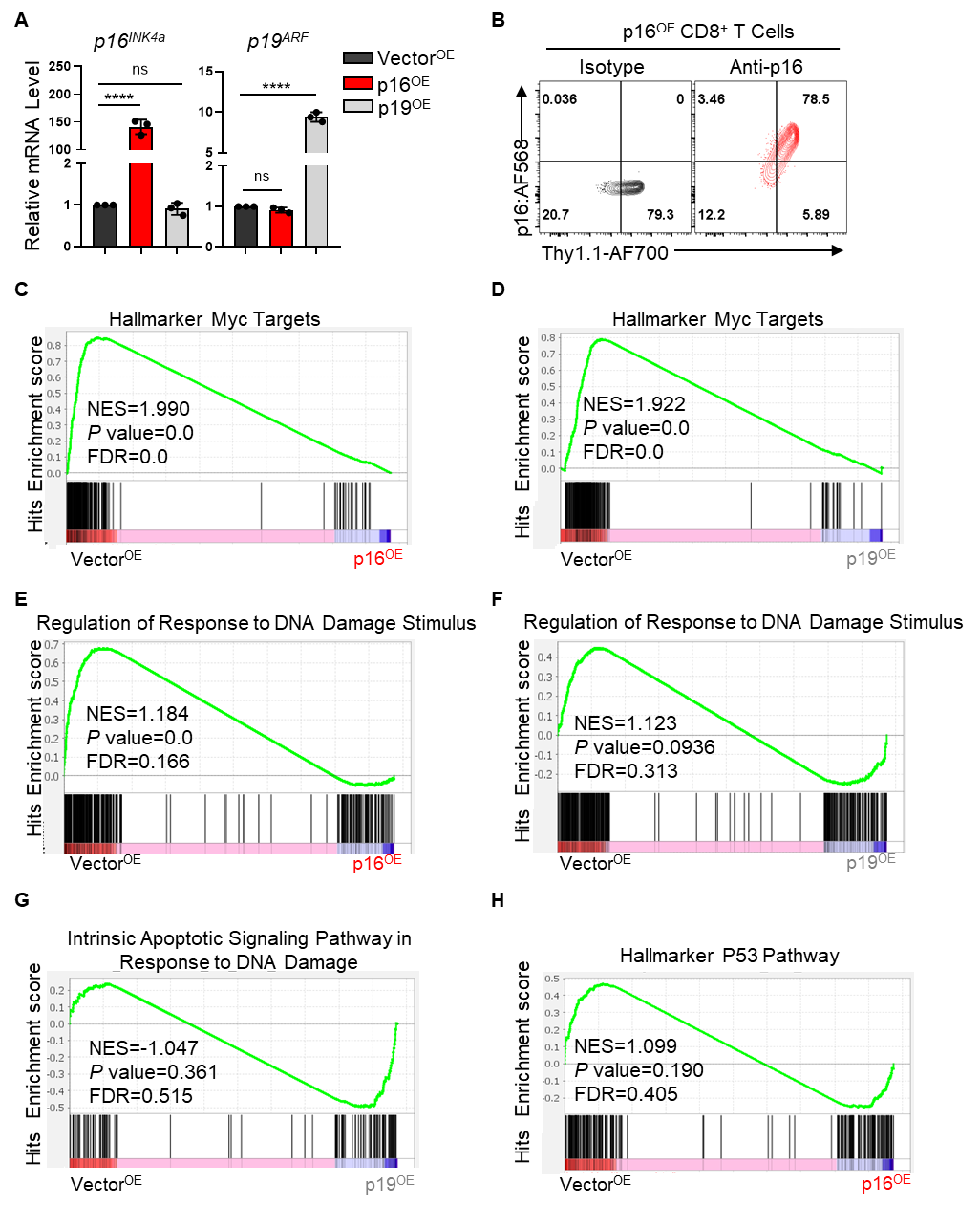


**Figure S2. p16 or p19 overexpression mediated transcription reprograms of the OT-1 T cells**

(**A**) qPCR experiments showed the relative mRNA levels of *p16^INK4a^* and *p19^ARF^* in Vector^OE^, p16^OE^ or p19^OE^ OT-1 T cells. Data are shown as Mean ± SEM (n=3), One-way ANOVA , *****: p＜0.0001;* ns: no significant. (**B**) Representative FACS plots of Thy1.1 and p16 expressions in p16^OE^ CD8^+^ T cells. (**C-H**) GSEA analyses of Myc target genes, regulation of response to DNA damage stimulus and response to DNA damage associated genes which obtained from Vector^OE^ compare to p16^OE^ or Vector^OE^ compare to p19^OE^ enriched CD8^+^ T cells.


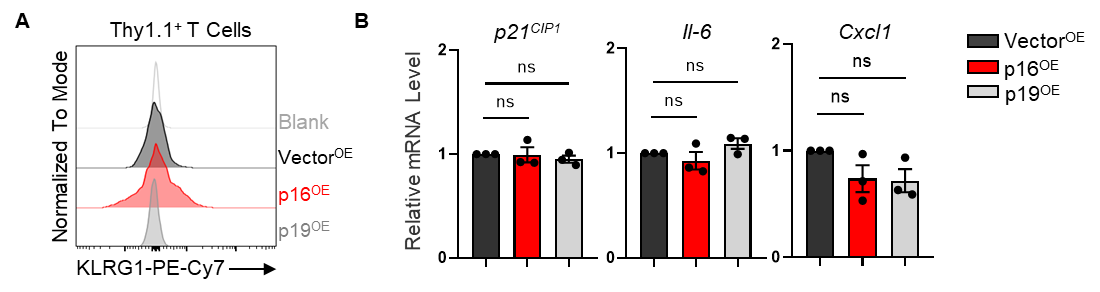


**Figure S3. The senescence phenotypes of p16^OE^ or p19^OE^ cells**

(**A**) Representative histogram of KLRG1 in Vector^OE^, p16^OE^, p19^OE^ CD8^+^ T cells. (**B**) qPCR experiment showed the relative mRNA levels of *p21^CIP1^*, *Il-6* and *Cxcl1* in p16 or p19 overexpressing OT-1 cells. Data are shown as Mean ± SEM (n=3), One-way ANOVA, ns: not significant.


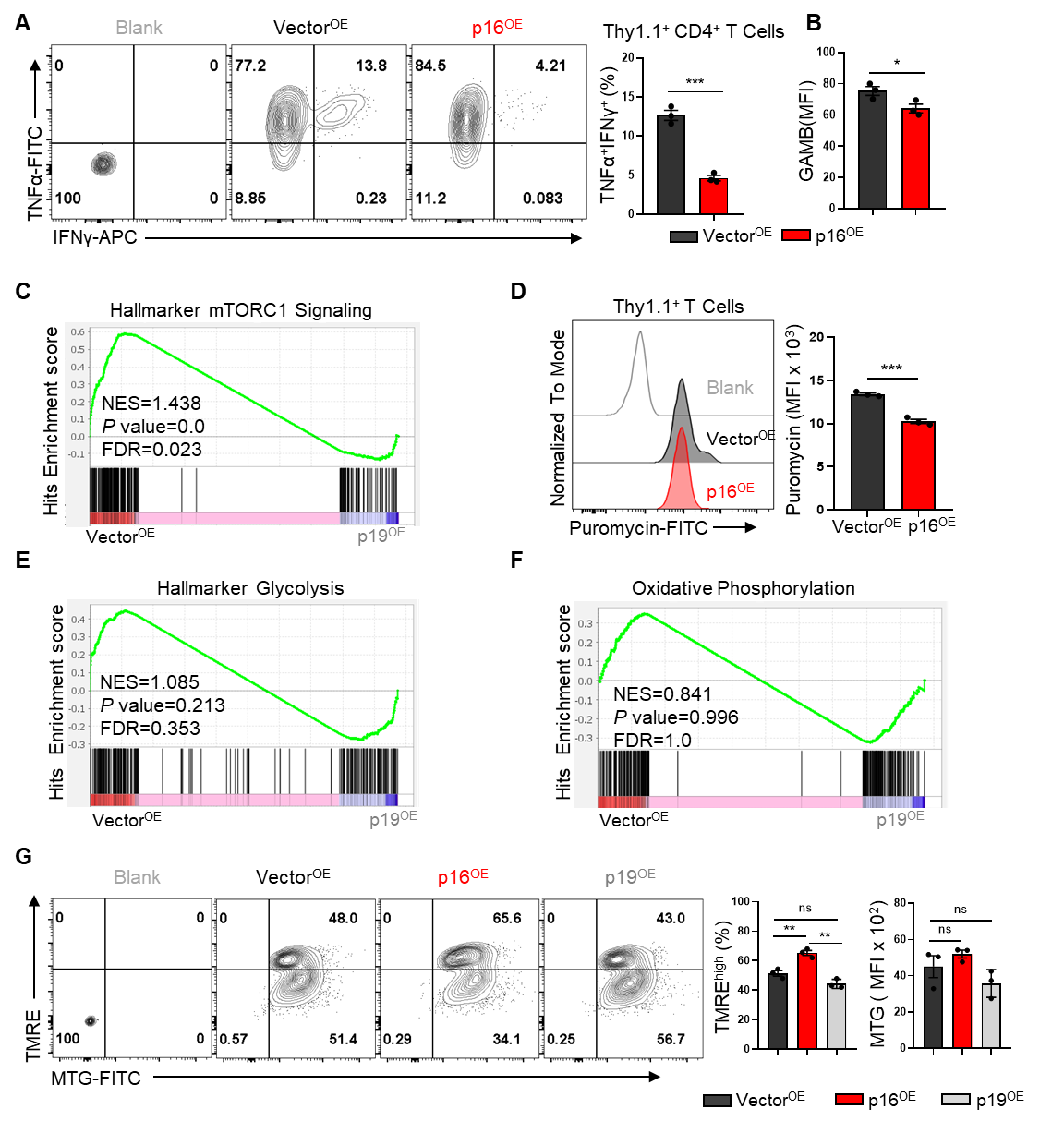


**Figure S4. Metabolic alternation of p16 or p19 overexpressed cells.**

(**A** and **B**) Left: representative contour plots and summary of p16 overexpressing CD4^+^ T cells producing cytokines TNF-α, IFN-γ. Right: summary of the levels of GzmB MFI in vector or p16 overexpressing CD4^+^ T cells. Data are shown as Mean ± SEM (n=3), One-way ANOVA, **: p < 0.05*; ****: p < 0.001*. (**C, E** and **F**) mTORC1, Glycolysis and Oxidative phosphorylation associated gene sets enrichment analysis performed on RNA-seq data which obtained from Vector^OE^ compared to p19^OE^ enriched CD8^+^ T cells. (**D**) Left: representative histogram of puromycin levels in indicated cells. Right: statistical analysis of puromycin MFI in these two groups. Data are shown as Mean ± SEM (n=3), Student’s t test, ****: p < 0.001*. (**G**) Left: representative contour plots of TMRE and MTG showing mitochondrial membrane potential and mass in p16 or p19 overexpressing OT-1 cells. Right: The percentage of TMRE^high^ and MTG MFI were compared in three groups. Data are shown as Mean ± SEM (n=3), One-way ANOVA, ***: p < 0.01*; ns: not significant.


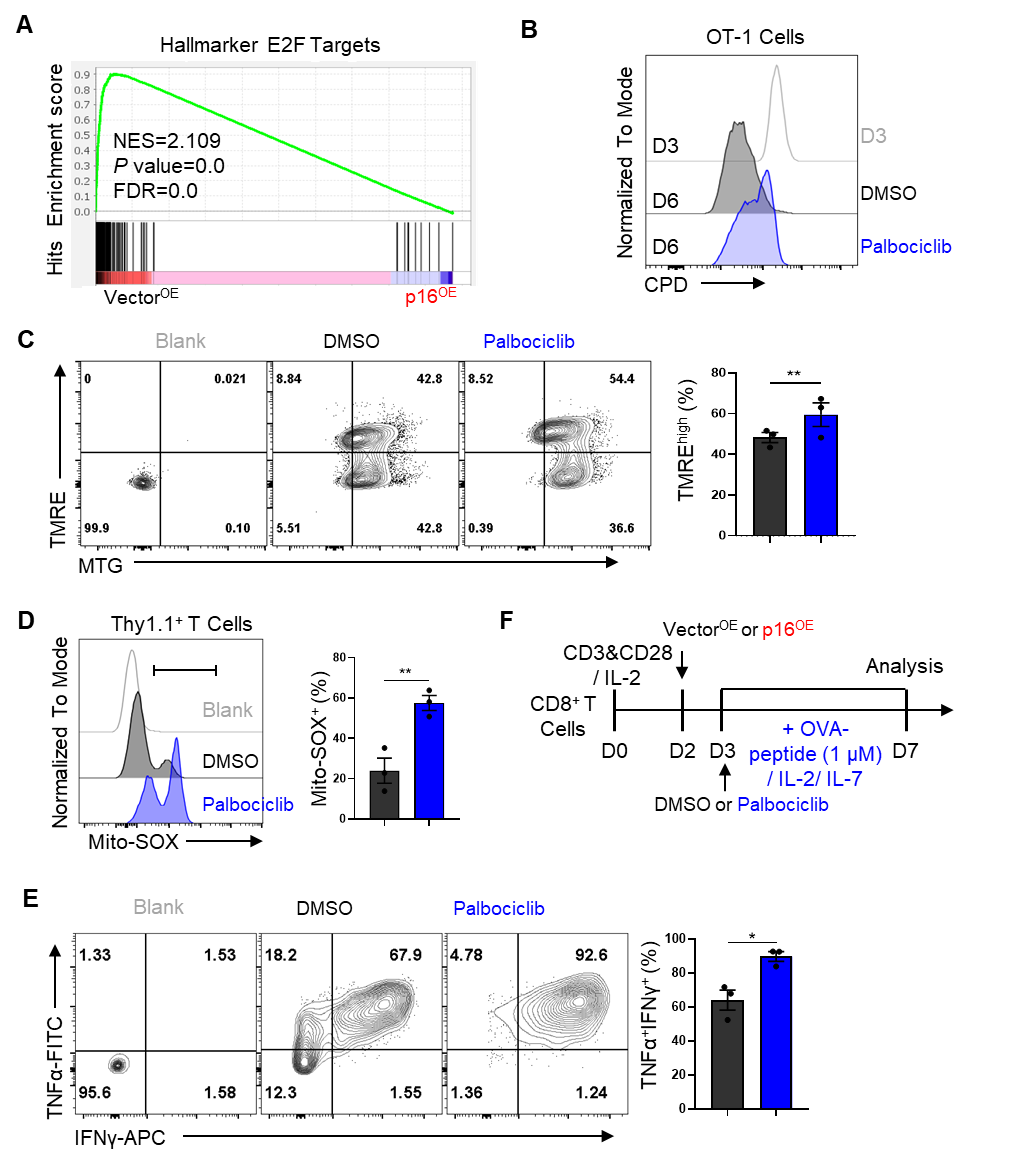


**Figure S5. CDK4/6 inhibitors arrest CD8^+^ T cell cycle**

(**A**) E2F targets Gene set enrichment analysis performed on RNA-seq data which from Vecotr^OE^ compare to p16^OE^ enriched CD8^+^ T cells. (**B**) Naïve OT-1 cells were labeled by CPD 450, and activated by αCD3/CD28 3 days. Then activated cells are treated with DMSO or Palbociclib, flow cytometry analyzed the proliferation index of the indicated genotypes 3 days later. (**C**) Left: representative contour plots of TMRE and MTG showing mitochondrial membrane potential and number in DMSO or Palbociclib-treated OT-1 cells. Right: percentage of TMRE^high^ were compared. (**D**) The mitochondria redox levels in OT-1 cells with indicated treatment were marked by Mito-SOX (right), and then percentages of Mito-SOX^+^ in two groups were compared (left). (**E**) Left: the ability of cytokine production of DMSO or Palbociclib-treated OT-1 cells were analyzed by flow cytometry. Right: percentage of TNF-α^+^IFN-γ^+^ frequencies in the indicated cells. Data are shown as Mean ± SEM (n=3), Student’s t-test, **: p＜0.05*; ***: p＜0.01*. (**F**) Diagram of the experimental strategy used in persistent antigen stimulation model.


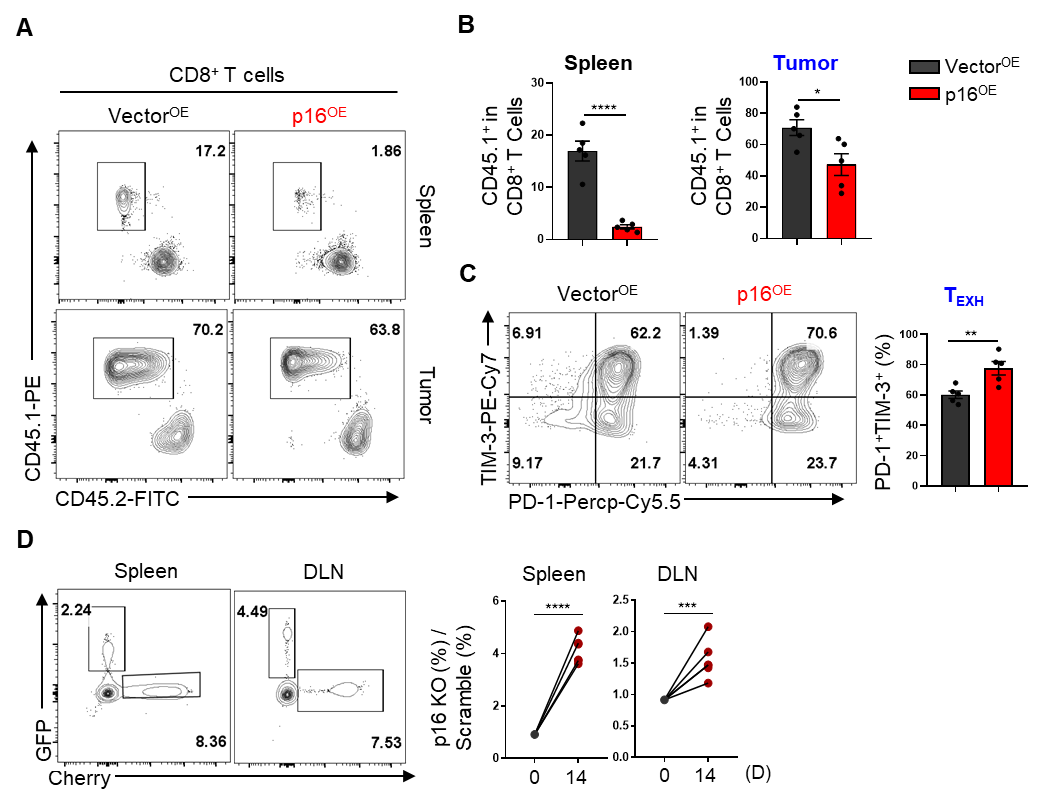


**Figure S6. High expression of p16 reduced the anti-tumor ability of adoptively transferred OT-1 cells**

(**A**) Representative FACS plots of Vector^OE^ or p16^OE^ OT-1 cells in total OT-1 cells of recipient mice spleen and tumor. (**B**) Summary of the transferred OT-1 cells frequency in recipient mice spleen and tumor. Data are shown as Mean ± SEM (n=5), Student’s t test, **: p＜0.05; ****: p＜0.0001*. (**C**) Left: representative contour plots of PD-1 and TIM-3 expression in indicated OT-1 TILs. Right: the percentage of OT-1 TILs in the PD-1^+^TIM-3^+^ T_EXH_ subset. Data are shown as Mean ± SEM (n=5), Student’s t test, ***: p＜0.01*. (**D**) Representative FACS plots (left) and kinetics (right) Scramble and p16 KO OT-1 cells in recipient mice spleen and DLN at 14 days post-i.v., respectively. Data are shown as Mean ± SEM (n=5), Student’s t test, ***: p＜0.01*; ****: p＜0.001*.
