# Supplementary figures and images for: The cell cycle regulator p16 promotes tumor infiltrated CD8+ T cell exhaustion and apoptosis
Source: Cell Death Dis. 2024 May 15;15(5):339. doi: 10.1038/s41419-024-06721-7 (PMC11096187; doi:10.1038/s41419-024-06721-7)

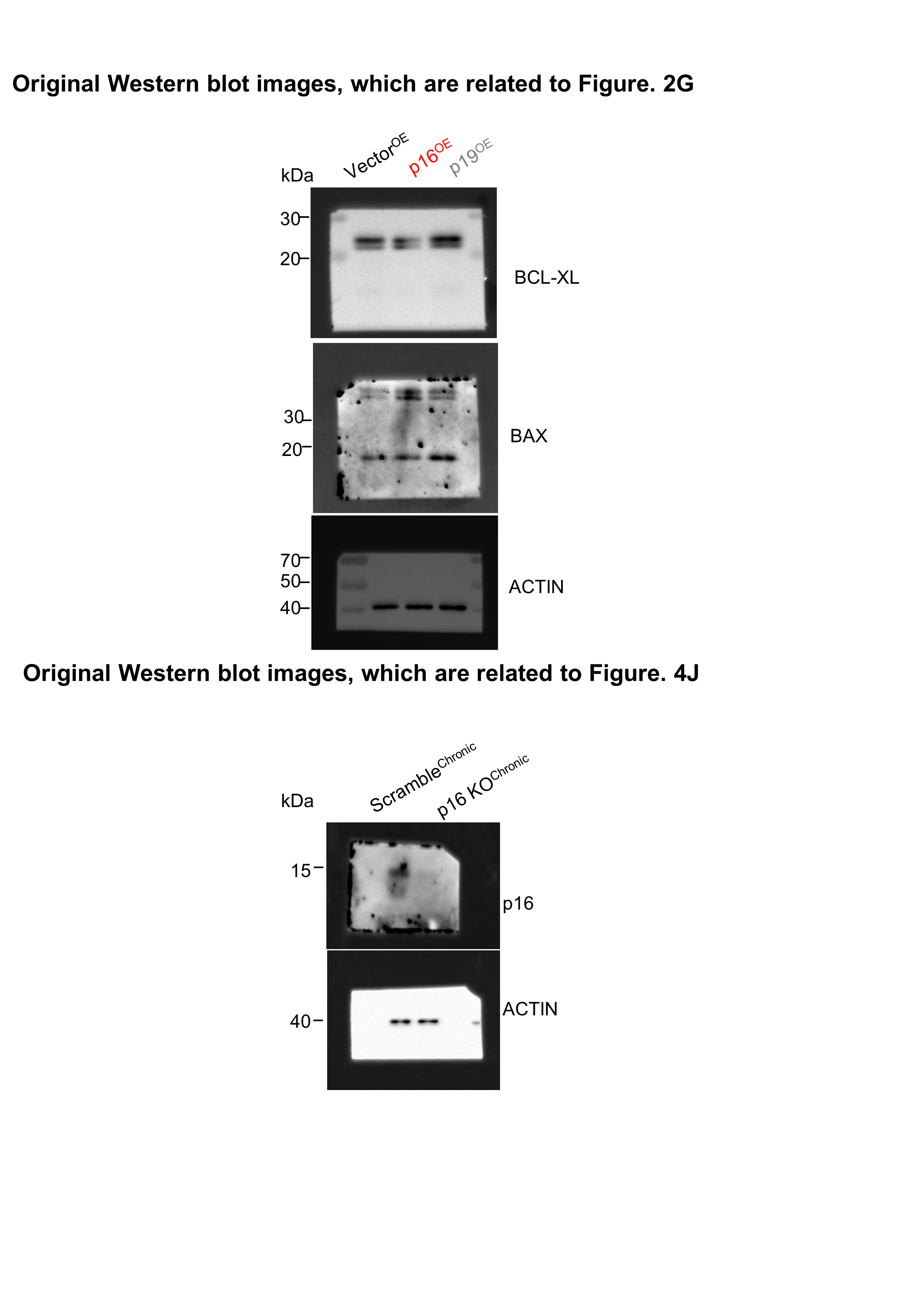

Supplement: Supplementary file 2 — Original Western blot images [file 41419_2024_6721_MOESM2_ESM.tif]
